# Supplementary material for: The effects of genital myiasis on the diversity of the vaginal microbiota in female Bactrian camels
Source: BMC Vet Res. 2022 Mar 5;18:87. doi: 10.1186/s12917-022-03189-5 (PMC8897907; doi:10.1186/s12917-022-03189-5)
Supplement: Supplementary file 5 — Additional file 5. [file 12917_2022_3189_MOESM5_ESM.zip › MPL201709200_16s_yy/Treat1/C07_picrust/KEGG_L2_Unclassified.pdf]

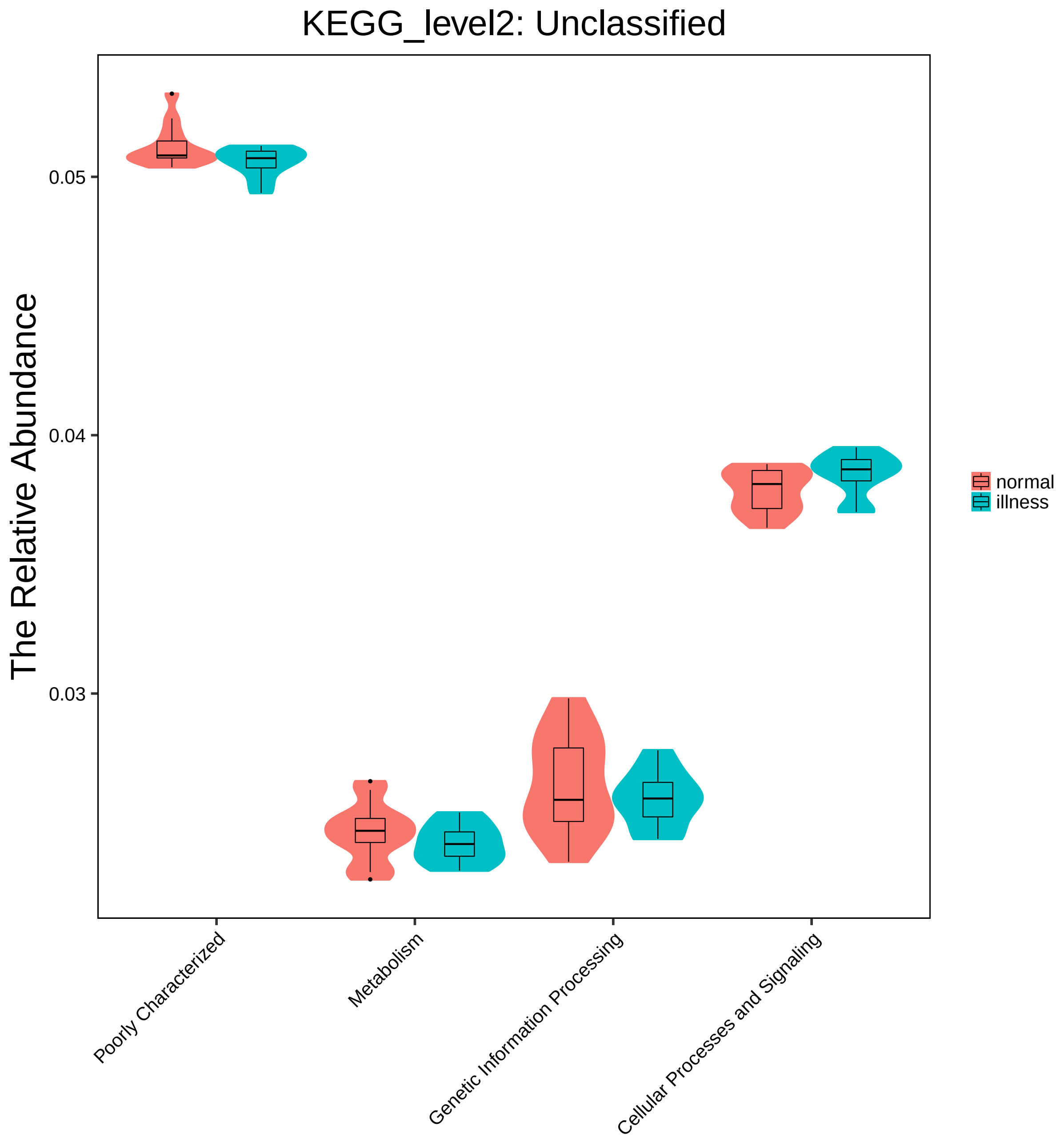

KEGG_level2: Unclassified
The Relative Abundance
0.05
0.04
0.03
Poorly Characterized
Metabolism
Genetic Information Processing
Cellular Processes and Signaling
normal
illness
